# Aripirazole-Long Acting Injectable in Pregnant Women with Schizophrenia: A Case Series

**DOI:** 10.1192/j.eurpsy.2022.355

**Published:** 2022-09-01

**Authors:** B. Fernández-Abascal, M. Recio-Barbero, M. Saenz-Herrero, R. Segarra

**Affiliations:** 1 University Hospital Marqués de Valdecilla, IDIVAL Hospital, Department Of Psychiatry, Santander, Spain; 2 Cruces University Hospital, Biocruces Vizcaya Health, Barakaldo, Spain; 3 Cruces University Hospital, Departament Of Psychiatry, Barakaldo, Spain

**Keywords:** Long-acting injectable antipsychotics, Pregnancy, schizophrénia, second-generation antipsychotics

## Abstract

**Introduction:**

Long-acting injectable antipsychotics (LAIs) have emerged as a new therapeutic option to treat patients suffering a psychotic disorder. To date, there is a lack of studies regarding safety and clinical use pattern of LAIs in pregnant women.

**Objectives:**

Provide evidence and real world clinical data of pregnant women with schizophrenia who have been treated with long-acting aripiprazole monohydrate (aripiprazole once monthly [AOM] condition) during their pregnancy.

**Methods:**

Descriptive real-world clinical experiences of pregnant women in treatment with AOM. The information was obtained by reviewing electronic medical records and by direct clinical observation management.

**Results:**

The first six case-series describing the pregnancy course of women with schizophrenia treated with AOM. All of them remained psychopathologically stable through pregnancy, and their infants became healthy with normal developmental milestones (Table 1).

**Table 1.** Clinical characteristics of six case-reports.

Mothers	1	2	3	4	5	6
Maternal/Pregnancy outcomes						
Age(years)	35	29	35	31	38	30
Diagnosis	Schizophrenia	Schizophrenia	Schizophrenia	Schizophrenia	Schizophrenia	Schizophrenia
AOM(mg/days)	400-300	400-300	400-300	160	300	400
Type of delivery	Eutocic.	Eutocic, preterm	Eutocic	Eutocic	Eutocic	Eutocic
Neonatal outcomes						
Weight(grams)	3300	1800	3140	3102	2940	3400
Gender	Female	Female	Male	Male	Male	Male
Developmental Abnormalities (years)	No(3)	No(2)	No( 0.17)	No(2)	No(2)	No(1.5)

**Conclusions:**

The favorable results in this case-series suggest that despite the lack of evidence on reproductive safety and treatment with AOM during pregnancy, this therapeutic option should be considered in pregnant women with schizophrenia. However, further research on the use of long-acting antipsychotics in pregnant women is needed.

**Disclosure:**

No significant relationships.

